# A Game-Theoretic Response Strategy for Coordinator Attack in Wireless Sensor Networks

**DOI:** 10.1155/2014/950618

**Published:** 2014-07-01

**Authors:** Jianhua Liu, Guangxue Yue, Shigen Shen, Huiliang Shang, Hongjie Li

**Affiliations:** ^1^College of Mathematics, Physics and Information Engineering, Jiaxing University, Jiaxing 314001, China; ^2^Department of Electronic Engineering, Fudan University, Shanghai 200433, China

## Abstract

The coordinator is a specific node that controls the whole network and has a significant impact on the performance in cooperative multihop ZigBee wireless sensor networks (ZWSNs). However, the malicious node attacks coordinator nodes in an effort to waste the resources and disrupt the operation of the network. Attacking leads to a failure of one round of communication between the source nodes and destination nodes. Coordinator selection is a technique that can considerably defend against attack and reduce the data delivery delay, and increase network performance of cooperative communications. In this paper, we propose an adaptive coordinator selection algorithm using game and fuzzy logic aiming at both minimizing the average number of hops and maximizing network lifetime. The proposed game model consists of two interrelated formulations: a stochastic game for dynamic defense and a best response policy using evolutionary game formulation for coordinator selection. The stable equilibrium best policy to response defense is obtained from this game model. It is shown that the proposed scheme can improve reliability and save energy during the network lifetime with respect to security.

## 1. Introduction

Both the security and the quality of service (QoS) of ZWSNs are important factors which will affect various services of sensor data delivery, for example, environmental monitoring [1], building monitoring to assess earthquake damage [2], and intelligent home monitoring [3]. On one hand, QoS is one of the key factors for various services that include diverse important parameters, that is, delay, throughput, and dropping probability of packets. Different types of services always need different requirements of QoS. On the other hand, sensor transmission always faces many malicious attacks [4]. In order to get secure network, various security protocols are presented to address security issues [5], including wired equivalent privacy (WEP), 802.1X port access control with extensible authentication protocol (EAP), and IP security protocol (IPsec) [6].

An IEEE 802.15.4 WSN is composed of one coordinator and a set of nodes [7]. The network topology defined in the standard is called cluster tree, where nodes associate with coordinators to establish parent child relationships and form a tree rooted at coordinator. Existing ZWSNs can cooperate with each other to compose sensor service and provide effective and efficient data delivery.

It is evident that sensor services have a certain requirement on both security and QoS to get good performance. Since the requirements might be different in a different circumstance or time, it may be impossible to satisfy requirements from both security and QoS simultaneously due to limited network resources. Considering only the costs of transmission without taking into account the possibility of coordinator attacked is not sufficient for providing secure composition in these environments. To avoid single point of failures owing to attack and increase network lifetime, it is desirable that the service composition based on coordinator selection method executes in a distributed manner.

Wireless sensor data can be proactively received by a coordinator owned by the same network operator. However, when a coordinator node is no longer in the reliable state, the rate of data delivery and QoS will be poor. Therefore, to improve QoS, multiple sensor nodes can share the reliable coordinator in which case the cost of sensor data transmission for each sensor node will be reduced. In other words, multiple sensor nodes can form a coalition to share the coordinator. When the coordinator nodes in the same coalition are reliable owing to unattack, each of the sensors can fully access the coordinator. However, if one coordinator node from the same coalition is unreliable owing to heavy attack, coordinators failures can occur; therefore, a coordinator selection mechanism would be required for sensor data delivery in the same coalition. In this context, two key questions are (i) how to form coalitions among sensor nodes to share the reliable coordinator to minimize the cost of energy and (ii) how to defend attacker to meet the required QoS requirements. To answer these questions, a joint dynamic defense and coordinator selection scheme are proposed using game theoretical concepts.

Given that the sensors are rational to minimize their own cost, a game-theoretic model is developed to find a solution of the defense attack and coordinator selection problem. This game model consists of two interrelated formulations, that is, a stochastic game for dynamic attack response and evolutionary game for coordinator selecting. The stochastic game formulation utilizes the coalitional structure obtained from the evolutionary game, while the evolutionary game formulation utilizes the cost and QoS performance measures from the stochastic game.

In this paper, our main contributions can be summarized as follows.

- We propose a proactive scheme for defending networking coordinators. It enables the defender to proactively select reliable coordinator to minimize the expected network energy loss. To our best knowledge, this is the first work that considers defending and attacking from the perspective of games.
- We formulate the problem of the defending networking coordinators as a 2-player zero-sum game. The payoff of our problem is measured by the maximum sensor service network utility. We propose an evolutionary game-theoretic framework for the defense response policy in which nodes in the network are regarded as players and the local combination of estimation information from different neighbors is regarded as different strategies of coordinator selection.
- We propose a new state estimation algorithm for selecting coordinators using fuzzy logic. We prove that a global Nash equilibrium (NE) exists. We then design a mixed-strategy solution for the defender and attacker that combines the evolutionary game NE strategies and stochastic game NE strategies in order to achieve the maximum payoffs for both players.

The rest of this paper is organized as follows.  Section 2 describes related works.  Section 3 describes the system model.  Section 4 presents the stochastic game for dynamic defense and a best response policy using evolutionary game formulation for coordinator selection.  Section 5 performs numerical experiments; the influence of a cost parameter is illustrated.  Section 6 concludes the paper.

## 2. Related Works

ZWSNs security and the quality of service (QoS) combining deployment and management related topics have become an active research area. One of the major constraints of ZWSNs deployment and management is the limited energy. It is crucial for maximizing the lifetime of ZWSNs that data packets are routed to the destination in an energy-efficient manner [8]. ZWSNs are widely studied route metrics for the number of hops [9–11]; hierarchical protocols [12, 13] group nodes into clusters and energy expenditure [14–17]. The relationship between number of hops and network energy for a single packet is investigated in [18]. Most of these approaches do not consider the reliability on the coordinator nodes that suffer attack from malicious nodes. In this paper, we study the possibility of using game theoretical approach to defend malicious nodes. To combat the attack on relay, several lightweight authentication protocols, which are based on computationally efficient hash chain, can be applied in cooperative wireless communication networks. Timed efficient stream loss tolerant authentication (TESLA) is a broadcast authentication protocol based on loose time synchronization [19]. Law et al. [20] showed how the jamming can be used to perform attacks on the network link layer protocols. Xu et al. [21] surveyed issues related to performing a jamming attack against sensor networks by examining both the attack and defense; they presented the following jamming models: constant jammer, deceptive jammer, random jammer, and reactive jammer.

Yao et al. [22] proposed a parameterized and localized trust management scheme for WSN security, particularly for secure routing, where each node only maintains highly abstracted parameters to evaluate its neighbors. Aivaloglou and Gritzalis [23] proposed a hybrid trust and reputation management protocol for WSNs by combining certificate-based and behavior-based trust evaluations. Gabrielli et al. [24] analyzed the security vulnerabilities of PEAS, ASCENT, and CCP and represented securing topology maintenance protocols. Bao et al. [25] considered multidimensional trust attributes derived from communication and social networks to evaluate the overall trust of a sensor node. Zonouz et al. [26] employed a game-theoretic response strategy against adversaries modeled as opponents in a two-player Stackelberg stochastic game and proposed fuzzy logic theory to calculate the network-level security metric values. However, [24–26] do not provide response strategy against attackers for coordinators.

Game theory provides a rich set of tools that can be used to model the attack behavior of malicious nodes. Game theory models were applied to solve various issues in wireless networks. In [27], a stochastic game was formulated for network selection problem in cognitive heterogeneous networks. In [28], an evolutionary game model was used to analyze the information diffusion process and the filtering over the adaptive networks. In this paper, we use stochastic and evolutionary game theory to study the cooperative defense behavior of sensors for coordinator selection under security constraints.

## 3. System Model

### 3.1. ZWSN Functionality and QoS

**Figure 1:**
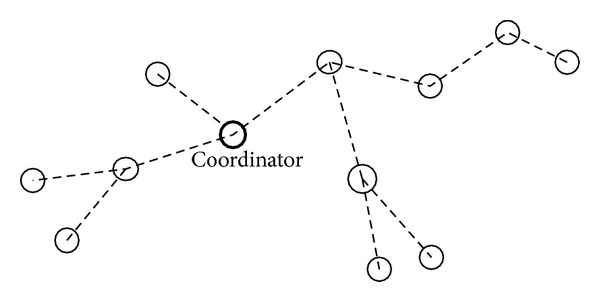
Selecting coordinator coalitional game model.

Each node typically provides a basic functionality for operating on the monitored data; sensors provide their functionalities through receiving and forwarding operations. That is, a data packet comes to a sensor, and the sensor receives and forwards to coordinator, while the network of sensor nodes collectively provides a composite service to the end nodes. Unlike the web environment where service provider availability and ample communication bandwidth are typically assured, sensor networks are highly dynamic as nodes often fail or become disconnected and wireless communication capacity is limited. The operation of every ZWSN is associated with two QoS attributes: the average number of hops to the coordinator (hop for minimization) and energy cost (energy for minimization). Energy cost is defined as the fee that a ZWSN consumer has to pay to receiving and forwarding operations. Hop is defined as the total length of path covered between the source of the sensor and the coordinator.  Figure 1 illustrates the forwarding and selecting coordinator coalitional game model. Cluster coalition can communicate with other coalitions by coordinators. Not only can a coordinator request or be invited to join a coalition, but also it can leave a coalition. Every coalition has a coordinator and a set of sensors as its members. The coordinator is responsible for receiving and forwarding data and managing the cooperation among these members.

In a coalition, coordinator is the controller of the network and it is responsible for initiating the network set-up; it starts by selecting a suitable communication channel. This selection is performed by the energy detection scan which assesses the level of interference on each channel by measuring the peak energy on each available channel. If a node is available, it selects a node to join coalition; this node starts an exchange of signaling packets with the chosen coordinator to complete formation coalition. A cooperation coalition is managed by the coordinator, which is a sensor composition *S* = [*s*[1],…, *s*[*n*]] of the coalition members {1,…, *n*}; once the coordinator receives a sensor's request, it forwards *s*[1]. If *s*[1] is available, the coordinator will send data to *s*[1]. Otherwise, it will forward *s*[2]. This process continues until a sensor is available and data is delivered to destination node. Suppose sense *i*'s availability is *a*_*i*_. Clearly, the resource availability of the coalitions *M* is given by

(1) $$\begin{array}{c} a_{r}=1-\overset{n}{\underset{i=1}{\prod }}\left(1-a_{i}\right). \end{array}$$

As a coalition can be regarded as a composite sensor, it also possesses other performance metrics as discussed earlier. In particular, its price *p*_*M*_ relies on its availability. Besides, following the widely accepted assumption that the cost of a composite sensor totally depends on the cost of the average number of hops to the coordinator and energy cost, the cost of a coalition *M* is given by

(2) $$\begin{array}{c} c_{M}\left(s\right)=\overset{n}{\underset{i=1}{\sum }}\overset{i-1}{\underset{j=1}{\prod }}\left(1-a_{s[j]}\right)c_{s[i]}, \end{array}$$

where *c*_*s*[*i*]_ is the cost of coalition *s*[*j*]. Let *h*_*i*_ be the average number of hops from the node *k* to the coordinator. The energy cost for a single packet can be calculated as follows: *c*_*s*[*i*]_ = (*E*_*r*_ + *E*_*f*_) × *h*_*i*_ × *N*_*i*_, where *E*_*r*_ and *E*_*f*_ are energy of the receiving and forwarding, respectively. *N*_*i*_ is the number of nodes in coalition *i*. The value of a coalition *M* is a sensor composition *S* given by *v*_*M*_(*s*) = *p*_*M*_ × *a*_*M*_ − *c*_*M*_(*s*).

### 3.2. Stochastic Game for Coordinator Attack

In this section, we present the game-theoretic formulation of the self-organized network selection problem. We model the coordinator attacked problem as a noncooperative game where the malicious sensors are the attackers, the coordinator sensors are the defenders, and the coordinator run state is considered as the internal state (*NormalState* (NS) or* HackedState* (HS)). The game is represented as

(3) $$\begin{array}{c} G=\left(N,Z,{\left\{A_{k}\right\}}_{k\in N},{\left\{u_{k}\right\}}_{k\in N}\right), \end{array}$$

where *N* is the set of players, *N* = {1,2} = {attacker, defender}.

*Z* is the space of states, *Z*_*i*_ = {NS, HS}, and  {*A*_*i*_}_*i*∈*N*_ is the set of actions (attack and defense) that player *i* can take. *A*_*i*_ = {*a*_*i*_, *r*_*i*_, *d*_*i*_, *∅*_*i*_};*a*_*i*_ is the attack action bringing the coordinator from state* NormalState* to* HackedState*. *r*_*i*_ is the resignation of the attack in state* NormalState*. *d*_*i*_ represents that the attack action *a*_*i*_ will be detected by the defender. *∅*_*i*_ represents that the attack *a*_*i*_ action will be undetected.

{*u*_*i*_}_*i*∈*N*_ is the utility function of player *i*. The defenders aim at maximizing the network lifetime with carefully-designed coordinator selecting schedules, while the malicious attackers want to decrease the network lifetime by strategic jamming. Therefore, they have opposite objectives and their dynamic interactions can be well modeled as a noncooperative (zero-sum) game. The coordinators as defenders are cooperative and rational players with the objective of maximizing their network throughput and decreasing the average number of hops and energy cost to itself. Thus, we define the utility function as defender's expected payoff for a choice of action as

(4) $$\begin{array}{c} u_{i}\left(a_{i},a_{-i}\right)=E\left[v_{i}\left(s\right)\;|\;\left(a_{i},a_{-i}\right)\right]=\underset{a\in A}{\sum }p\left(a\right)v_{k}\left(a\right). \end{array}$$

The choice of defender *i* that maximize defender *i*'s expected payoff over its action space *A*_*k*_ is called the player's best response action. The decision making of defender *i* in a game then becomes

(5) $$\begin{array}{c} \left(G\right):\underset{a_{i}\in A_{k}}{\max}\;u_{i}\left(a_{i},a_{-i}\right),\;\forall i\in N. \end{array}$$

### 3.3. Best Response Policy Using Evolutionary Game Formulation

Given a network topology denoted as a directed graph *G* = (*N*, *E*), *N* is the set of nodes and *E* is the set of arcs. Each node *i* ∈ *N* has the initial resource availability *a*_*i*_. Let *r*_*i*_ be the self-resistance of node *i* to attacks. Let *x*_*i*_ and *y*_*i*_ be the defending and attacking resource allocated to defenders and attackers, respectively. We adopt the contest model proposed in [29] where the resource availability loss ratio of node *i* is given by

(6) $$\begin{array}{c} \tau_{ij}\left(x_{i}\right)=\frac{{\left(y_{i}\right)}^{m}}{\alpha_{i}{\left(r_{i}+x_{i}\right)}^{m}+{\left(y_{i}\right)}^{m}}, \end{array}$$

where *m* ∈ (0,1] reflects the nonlinearity or linearity of the loss ratio on node *i*, and *α*_*i*_ is a parameter reflecting the relative difficulties for the defender to protect in a particular node compared with the attacker. When *α*_*i*_ ∈ (0,1), the defender has to allocate more resources than the attacker in order to mitigate the effect of the attack, while *α*_*i*_ > 1 means that the defender can easily detect and mitigate the effect of the attack. The payoff of a coalition *M* is a sensor composition *S* rewritten as

(7) $$\begin{array}{c} v_{M}^{a}\left(s\right)=p_{M}\times a_{M}-c_{M}\left(s\right)-\tau_{M}\left(s\right). \end{array}$$

Let *A*_0_ be the original sensor composition *S* resource availability without suffering from any attack. For the defender, its goal is to maximize the payoff *v*_*M*_^*a*^(*s*) over *x* to protect the maximum network QoS by selecting coordinator as much as possible. On the other hand, the attacker aims to minimize *v*_*M*_^*a*^(*s*) by attacking key coordinator nodes or, equivalently, maximize *A*_0_ − *v*_*M*_^*a*^(*s*) over *y*. Equation (7) suggests that *v*_*M*_^*a*^(*s*) can be increased if *c*_*M*_^*a*^(*s*) can be reduced. It is to be noticed that *c*_*M*_(*s*) depends on *h*_*i*_, *E*_*r*_, and *E*_*f*_; a reduction of *h*_*i*_, *E*_*r*_, and *E*_*f*_ also reduces *c*_*M*_(*s*). This can be obtained by evolutionarily selecting the coordinator position and deciding its reliability state. For coordinator node *i* with degree *d*_*i*_ (not including node itself) and coalition set {*i*_1_,…, *i*_*d*_*i*__}, the general parameter updating rule can be written as

(8) hi,t+1=Bi,t+1(Φ(hi1,t),Φ(hi2,t),…,Φ(hidi,t))=∑l∈w∑j∈NBi,t+1(j,l)Φ(hj,l,t),

where Φ(·) can be any adaptive role configuration function. *B*_*i*,*t*+1_ represents some specific linear combination rules, *w* represents the number of coalitions, and *N* denotes the number of coalitions members.

Evolutionary game theory (EGT) is originated from the study of ecological biology [30], which differs from the classical game theory by emphasizing more on the dynamics and stability of the whole population's strategies, instead of only the property of the equilibrium. Such an equilibrium strategy is defined as the evolutionarily stable strategy (ESS).

Let us consider an evolutionary game with *k* strategies *θ* = {1,2,…, *k*}. The utility matrix *U* is a matrix *k* × *k*, whose entries *γ*_*ij*_ denote the payoff for strategy *i* versus strategy *j*. The population fraction of strategy *i* is given by *p*_*i*_, where 0 < *p*_*i*_ < 1, *i* ∈ {1,…, *k*}. The fitness of strategy is given by *f*_*i*_ = ∑_*j*=1_^*k*^ *p*_*j*_ *γ*_*ij*_. For the average fitness of the whole population, we have *η* = ∑_*i*=1_^*k*^ *p*_*i*_ *f*_*i*_. The Wright-Fisher model has been widely adopted to let a group of players converge to the ESS [27], where the strategy updating equation for each player can be written as

(9) $$\begin{array}{c} p_{i}\left(t+1\right)=\frac{p_{i}\left(t\right)f_{i}\left(t\right)}{\eta \left(t\right)}. \end{array}$$

From (8), it can be seen that the strategy updating process in the evolutionary game is similar to the position parameter updating process in adaptive selecting of the coordinator problem. It is intuitive that we can use evolutionary game to formulate the distributed adaptive selecting of the coordinator position problem. Given the definition of the players, strategy space, and payoffs, the maximum network QoS of evolutionary game Ω can be defined as

- players: defender, attacker,
- strategy: *θ*_*ij*_,
- payoffs: *v*_*M*_^*a*^(*s*), *A*_0_ − *v*_*M*_^*a*^(*s*).

Each coordinator node represents a defense player; *θ*_*ij*_ denotes the probability that the strategy of node *i* will replace that of node *j* from its neighbor. We first discuss how players' strategies are updated in EGT, which is then applied to the position parameter updating in distributed adaptive coordinator selection. In EGT, the fitness of a player is locally determined from interactions with all adjacent players, which is defined as

(10) $$\begin{array}{c} f=\left(1-\lambda \right)\cdot v_{m}\left(s\right)+\lambda \cdot v_{M}^{a}\left(s\right), \end{array}$$

where *λ* parameter represents the selection of new coordinator node intensity, that is, the relative contribution of the game to fitness. The case *λ* → 0 represents the limit of weak selection of new coordinator node owing to jam weak attack, while *λ* = 1 denotes strong selection, where fitness equals payoff. There are two different strategy updating rules for the evolution dynamics called AC, TC.

**Figure 2:**
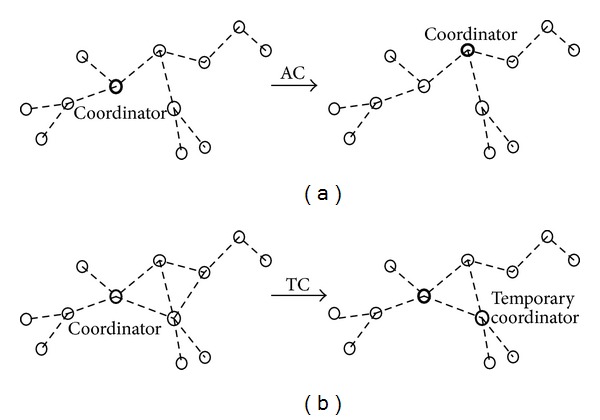
(a) Alternative coordinator. (b) Temporary coordinator.

- AC (alternative coordinator) update rule: a coordinator player is chosen to abandon his/her current coordinator role. Then, the chosen player selects one of its neighbors as coordinator with the probability of being proportional to their fitness; its neighbor copies its strategy and configuration, as shown in Figure 2(a).
- TC (temporary coordinator) update rule: a neighbor player adopts the strategy and configuration of one coordinator as a temporary coordinator node and remains with its current strategy; old coordinator configures as a temporary ordinary (TO) node, with the probability of being proportional to fitness. Moreover, the neighbor player keeps the strategy and configuration until the jamming attack weakens and old coordinator node recovers its role, as shown in Figure 2(b).

These two kinds of strategy updating rules can be matched to two different kinds of position parameter updating algorithms in distributed adaptive coordinator selection. The degree of coordinator node *i* is *d*_*i*_. We use *N* to denote the set of all nodes in a coalition.

For the AC update rule, the probability that the coordinator player selects and configures one of its neighbors *j* as coordinator is

(11) $$\begin{array}{c} P_{j}=\frac{f_{j}}{\sum_{q\in N}f_{q}}\frac{1}{\Gamma_{j}}, \end{array}$$

where the *f*_*j*_/∑_*q*∈*N*_ *f*_*q*_ is the probability that the neighboring node *j* is chosen to act as coordinator, and 1/Γ_*j*_ is the probability that node *j* is chosen for copying coordinator's strategy and updating configuration. The equivalent parameter updating rule for ZWSN can be written as

(12) hi,t+1=(fj∑q∈Nfq1Γj)ΦAC⁡(hj,t) +(1−fj∑q∈Nfq1Γj)∑i∈w∪N∖{j}Φ(hi,t),

where the first term is that the neighboring node is chosen for configuration as an alternative coordinator, and the second term is that all nodes are configured as a new average hop from source node to new coordinator.

For the TC update rule, the equivalent parameter updating rule for ZWSN can be written as

(13) hz,t+1=(fj∑q∈Nfp1Γj)ΦTC(hj,t) +(fj∑q∈Nfp1Γj)ΦTO(hi,t) +(1−fj∑q∈Nfp1Γj)∑z∈w∪N∖{j,i}Φ(hz,t),

where the first term is that the neighboring node is chosen for configuration as a temporary coordinator, the second term is that it itself is configured as a temporary ordinary node, and the third term is that all nodes are configured as a new average hop from source node to new temporary coordinator. The payoff of a coalition *M* is a sensor composition *S* rewritten by

(14) $$\begin{array}{c} v_{M}^{a}\left(s\right)=p_{M}\times a_{M}-c_{M}\left(s\right)-\tau_{M}\left(s\right)-c_{\Phi }\left(s\right), \end{array}$$

where *c*_Φ_(*s*) is the cost of configuration. Its immediate reward for a cluster sensor with *n* coordinators is defined as a weighted sum of the performance of a cluster sensor:

(15) $$\begin{array}{c} r_{\Phi }=\overset{n}{\underset{i=1}{\sum }}\left(w_{i}\cdot c_{\Phi }^{\mathrm{AC}}\left(i\right)+\left(1-w_{i}\right)\cdot \left(c_{\Phi }^{\text{TC}}\left(i\right)+c_{\Phi }^{\text{TO}}\left(i\right)\right)\right), \end{array}$$

where *w*_*i*_ < 1. The “goodness” of a configuration action in a given evolutionary state is measured by a value function *Q*(*Z*, *a*); we employ temporal-difference (TD) method for configuration function update:

(16) Q(Zt,at)⟵Q(Zt,at)+ω·[rΦ,t+1+ξ·Q(Zt+1,at+1)−Q(Zt,at)],

where *ω* is a learning rate parameter that facilitates convergence in the presence of stochastic transitions.

## 4. FQL Based Reinforcement Learning for Coordinator Selection 

### 4.1. Fuzzy Logic

Fuzzy logic is a mathematical approach to emulate human way of thinking and learning. Fuzzy systems have been used as function approximating to facilitate generalization in state space for generating continuous actions. We propose fuzzy Q learning (FQL) [10] to a fuzzy evolutionary game decision (FEGD) setting. The proposed FEGD takes into account the channel occupied information with respect to the coordinator (*C*_*o*_) and the amount of remaining battery energy of that coordinator (*E*_*b*_). Their degree of relevance is expressed as a function *χ* = *f*(*C*_*o*_, *E*_*b*_), where *χ* denotes “selection level” or “quality” of the coordinator. In FEGD system, the input linguistic parameters are the amount of channel occupied with coordinator (*C*_*o*_) and the amount of remaining battery energy of that coordinator (*E*_*b*_). The term sets for each input linguistic parameter are defined, respectively, as

(17) $$\begin{array}{c} T\left(C_{0}\right)=\left\{\text{Low}\left(\text{LO}\right),\text{High}\left(\text{HG}\right)\right\},\\ T\left(E_{0}\right)=\left\{\text{Low}\left(\text{LO}\right),\text{Moderate}\left(\text{ME}\right),\text{High}\left(\text{HG}\right)\right\}. \end{array}$$

The output linguistic parameter that is the possibility of coordinator selection is defined as

(18) $$\begin{array}{c} O\left(\chi \right)=\left\{\text{Low}\left(\text{LO}\right),\text{Moderate}\left(\text{ME}\right),\text{High}\left(\text{HG}\right)\right\}. \end{array}$$

The fuzzy rules matrix is also summarized in Table 1. Following FQL, we define fuzzy inference system for fuzzy evolutionary game as consisting of 4 rules of the following form.

1. IF *C*_0_ is HG AND *E*_0_ is HG THEN *χ* is HG.
2. IF *C*_0_ is HG AND *E*_0_ is ME THEN *χ* is ME.
3. IF *C*_0_ is LO AND *E*_0_ is ME THEN *χ* is ME.
4. IF *C*_0_ is LO AND *E*_0_ is HG THEN *χ* is ME.

There are a number of shapes that can be used for the membership function of each input such as trapezoidal and Gaussian shapes. We have chosen the Gaussian shape, since it is common in engineering applications and easy to use. A Gaussian fuzzy set membership degree in name is defined as follows:

(19) $$\begin{array}{c} \mu_{A}\left(x\right)=\exp\left(-\frac{{\left(x-\varepsilon \right)}^{2}}{2\sigma^{2}}\right), \end{array}$$

where *ε* and *σ* are the fuzzy number mean and standard deviation and are assigned initially, *μ*_*A*_ : *U* → [0,1], for instance, to quantify the rule shown in Table 1 for the input *f*(0.4,0.5), using (19) to calculate the *μ*_*A*_(0.4) = 0.14, *μ*_*A*_(0.5) = 0.01, *ε* = 0.2, and *σ* = 0.1. The main remaining part is how to quantify the logical “and” operation that combines the meaning of two linguistic terms into a single premise. Consider

(20) $$\begin{array}{c} f_{A}=\left(0.4,0.7\right)=\mu_{A}\left(0.4\wedge 0.5\right)=\min\left\{0.14,0.01\right\}=0.01. \end{array}$$

Finally, the numerical result of this fuzzy operation, defuzzification, is the last step in the operating procedure of the fuzzy inference mechanism; we use the most common method called a center of gravity. This method converts the fuzzy set into the value for which the area under the graph of the membership function, *χ* = *f*(*C*_*o*_, *E*_*b*_), is given by computing the center of gravity (CoG) of the area at the center:

(21) $$\begin{array}{c} \mu_{A}^{*}=\frac{\sum_{i=1}^{n}\mu_{A}\left(x_{i}\right)\times x_{i}}{\sum_{i=1}^{n}\mu \left(x_{i}\right)}. \end{array}$$

### 4.2. Stochastic Learning Procedure

Here, we discuss obtaining the NE via stochastic evolutionary learning. As the attacker strategy is time-varying and the defense action is selected by each player simultaneously. We propose a decentralized algorithm based on stochastic evolutionary learning (SEL), by which the coordinator learn toward the equilibrium strategy from their individual action-reward history.

To facilitate the development of the SEL-based algorithm, let the mixed strategy *P*_*i*_(*t*) = [*p*_*i*,1_(*t*),…, *p*_*i*,*M*_(*t*)] the coordinator selection probability vector for player *i*, where *p*_*i*,*a*_*i*__(*t*) is the probability that player *i* selects strategy *a*_*i*_ ∈ *A*_*i*_ at time *t*. The proposed self-organized defense algorithm by selecting coordinator is described in Algorithm 1.

**Algorithm 1 .**

Self-organized defense by selecting coordinator (SoDSC):

1. Initially, set *t* = 0 and the coordinator selection probability vector as *p*_*i*,*a*_*i*__(*t*) = 1/Γ_*j*_.
2. At every time *t*, each player selects an action *a*_*i*_(*t*) as the outcome of a probabilistic strategy based on *P*_*i*_(*t*).
3. The coalitions receive the instantaneous reward *v*_*i*_(*t*) specified.
4. Each coordinator in coalitions updates its selection probability vectors according to the following rules:
5. CID = getcurrent_node (ID)
6. CRS = Get_CoordinatorResourceState (CID) using FQL coordinator selection.

*REPEAT*

 
*IF CRS* = HG
   
*h*_*i*,*t*+1_⟵*h*_*i*,*t*_ according to  (12)
   
*p*_*i*,*a*_*i*__(*t* + 1)⟵*p*_*i*,*a*_*i*__(*t*) + *ϖ* · *h*_*i*,*t*+1_(1_{*a*_*i*_=*d*_*i*_}_ − *p*_*i*,*a*_*i*__(*t*))
    (22) Q(Zt+1,at+1) ⟵Q(Zt,at)   +ω·(∑i=1n(wi·cΦAC⁡(i))+ξ·Q(Zt+1,at+1)      ·1{ai=ΦAC⁡}−Q(Zt,at)).

*ELSE, IF CRS* = ME

 
   
*h*_*z*,*t*+1_⟵*h*_*z*,*t*_ according to  (13)
   
*p*_*i*,*a*_*i*__(*t* + 1)⟵*p*_*i*,*a*_*i*__(*t*) + *ϖ* · *h*_*z*,*t*+1_(1_{*a*_*i*_=*d*_*i*_}_ − *p*_*i*,*a*_*i*__(*t*))
    (23) Q(Zt+1,at+1) ⟵Q(Zt,at)+ω·(∑i=1n((1−wi)·(cΦTC(i)+cΦTO(i)))              +ξ·Q(Zt+1,at+1)·1{ai=ΦTC∧TO}              −Q(Zt,at)).
(5) * UNTIL* value function converges,

where 0 < *ϖ* < 1 is the learning rate. 1_{·}_ is the indicator function. *h*_*i*,*t*+1_ or *h*_*z*,*t*+1_ is the normalized reward.

The instantaneous reward serves as a reinforcement signal so that a high reward brings a high probability in the next strategy update (Step 4). Also note that coordinator selection based on a probabilistic experiment (Step 2) might result in reconfiguration between different evolutionary rules in the beginning of the learning procedure. However, a stable long-term best response strategy for defending will be yielded after the learning period and the time required for convergence is a small fraction of the total operation time.

**Algorithm 2 .**

Get_CoordinatorResourceState (CID):

1. Initialization: *C*_*o*_,  *E*_*b*_.
2. Use (19) to calculate *μ*_*A*_(*C*_*o*_)'s and *μ*_*A*_(*E*_*b*_)'s for *T*(*C*_0_) and *T*(*E*_0_), respectively, given by (17) and (18).
3. Combine *T*(*C*_0_) and *T*(*E*_0_) using Table 1 to form *O*(*χ*).
4. Calculate the *μ*_*A*_(*C*_*o*_∧*E*_*b*_)'s *O*(*χ*) resulting from step 3 as *μ*_*A*_(*O*(*χ*)) = min⁡{*μ*_*A*_(*C*_*o*_), *μ*_*A*_(*E*_*b*_)}.
5. Calculate the output of defuzzification *χ* = *f*(*C*_*o*_, *E*_*b*_) = *μ*_A_* according to (21).
6. Return *O*(*χ*).

Algorithm 2 describes the proposed fuzzy logic-based coordinator resource state decision algorithm from the point of view of a single coordinator node *C*_*i*_. Moreover, we also assume that the relay *C*_*i*_ is able to read its battery level *E*_*b*,*i*_ and estimate *C*_*o*,*i*_ using the ACK message from the coordinator.

**Proposition 3 .**

The SoDSC Algorithm converges to NE whenthe learning rate *ϖ*, *ω* is sufficiently small.

**Proof**

Let limit *P* of the interpolated process satisfies the ODE [31] and consists of *N* probability vectors.

Let *P* ^*d*^ = (*P*_1_^*d*^,…, *P*_*N*_^*d*^) be the mixed strategy of all players, which are denoted by *P*_*ij*_^*d*^. Let  Θ(*P*_*i*_^*d*^) = *E*[*u*_*i*_^*e*^] and *Υ*(*P*_*j*_^*d*^) = *E*[Φ_*j*_^*c*^] be the expected response reward function of player *i* and the expected configuration function, respectively, over the mixed strategy *P* ^*d*^. Ψ also has the same number of mixed strategy which will be denoted as Ψ_*ij*_. The component equations of (12)–(17) are

(24) dpi,ai(t)dt=pi,ai(t)∑ai′pi,at′(t)[Θi(πi,P−i)−Θi(πi′,P−i)]dQz,aj(t)dt=Qz,aj(t)∑aj′qz,aj′(t)[Υj(πj,P−j)−Υi(πj′,P−j)]dΨij(Pd)dt=∑i∑j∂Ψij(Pd)∂pi,jdpi,ai(t)dtdQz,aj(t)dt=pi,ai(t)·pi,at′(t)·Θ(π,P)·Qz,aj(t) ·qz,aj′(t)·Υ(π,P)≥0,

where

(25) $$\begin{array}{c} \Theta \left(\pi ,P\right)=\Theta_{i}\left(\pi_{i},P_{-i}\right)-\Theta_{i}\left(\pi_{i^{\prime }},P_{-i}\right),\\ \Upsilon \left(\pi ,P\right)=\Upsilon_{j}\left(\pi_{j},P_{-j}\right)-\Upsilon_{i}\left(\pi_{j^{\prime }},P_{-j}\right). \end{array}$$

Θ(*π*, *P*) and *Υ*(*π*, *P*) always have the same sign and are greater than zero. While the convergence to an NE is guaranteed as *ϖ* → 0, *ω* → 0. A smaller value of *ϖ*, *ω* leads to a slower convergence rate. A proper value of *ϖ*, *ω* can be numerically determined to strike the desired tradeoff between the accuracy and the rate of convergence for practical operations of the algorithm.

## 5. Simulation

An extensive simulation evaluation of dynamic defense and response strategy is reported in this section. We carry out our experiments using the Network Simulator 2 version 2.34 tool, which is a simulator implementing physical and MAC layers of the IEEE 802.15.4 standard. We first show that dynamic defense and response strategy increases average throughput by selecting unattacked coordinator to form a new network topology, hence a new coordinator, starting from the initial IEEE 802.15.4/ZigBee cluster trees. We also show the network lifetime increase over the basic IEEE 802.15.4/ZigBee configuration when fuzzy logic and evolutionary game for the defense response policy are applied to a network with coordinators failures owing to heavy attacks.

This section shows a comparison, in terms of jamming attack for cluster trees; network topology is formed with fuzzy logic and evolutionary game. We consider a network scenario consisting of* N* nodes, randomly deployed in a square area. The nodes' transmission range is reported in Table 2. At the beginning of each experiment the initial coordinator is randomly selected.

**Figure 3:**
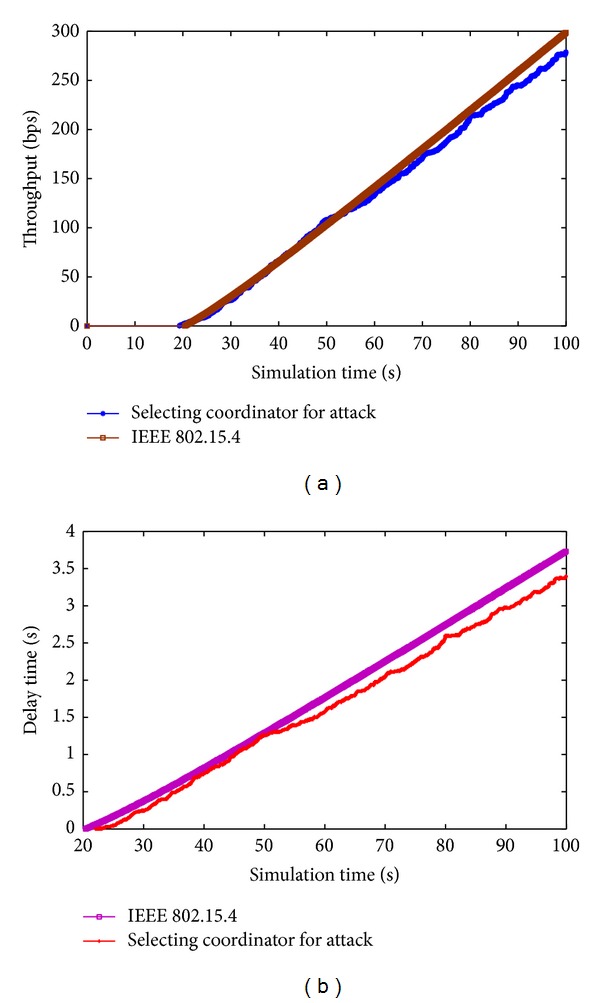
(a) The throughput related to the new topology for coordinator attacks. (b) The delay related to the new topology for coordinator attack.

As for the physical layer Network Simulator-2 implements all primitives described in the IEEE 802.15.4 Standard and uses the two-ray ground propagation model. Each packet received at the physical layer should be above the receive threshold value, that is assumed to be equal to 3.24 × 10^−10^ W to be correctly received. By varying the position of coordinator node and jamming attack on the field, we repeat the IEEE 802.15.4 association procedure for* N* times. At the end of the procedure we record the throughput related to the new topology for coordinator attack. Figures 3(a) and 3(b) show throughput and delay of defense for coordinator attack, respectively, and the results for IEEE 802.15.4 and selection of coordinator using Algorithm 1. When a network has a high jam attack, coordinator improves the topology configuration for defending jam attack by game selection; game selection has the same throughput and delay as IEEE 802.15.4 that is not selection of coordinator in face of jam attack. This is because the game selection Algorithm 1 uses TC and AC rules to select coordinator and keep higher throughput to approximate to IEEE 802.15.4 that does not face jam attack. Moreover, shorter routing paths can be established between any node and the coordinator, which saves node's energy and reduces data delivery delay.

**Figure 4:**
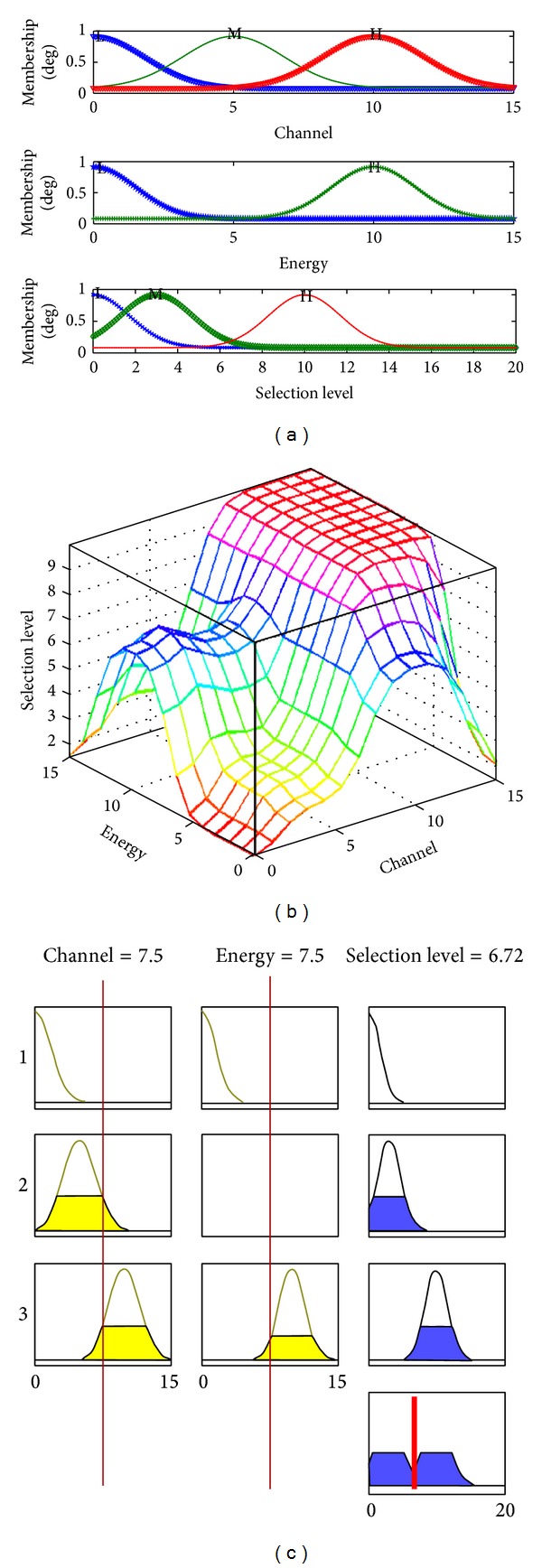
(a) Membership function for selection of coordinator. (b) The fuzzy inference for selection of coordinator. (c) The fuzzy inference process for selection of coordinator.

**Figure 5:**
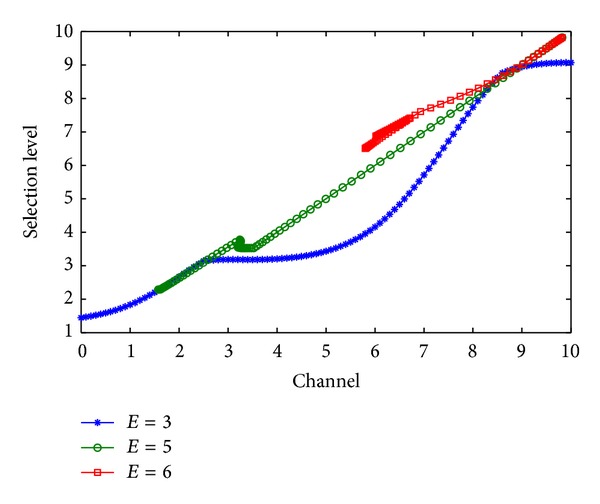
Effect of *E* = 3, *E* = 5, and *E* = 6 on the selection level for coordinator in face of jam attack.

**Figure 6:**
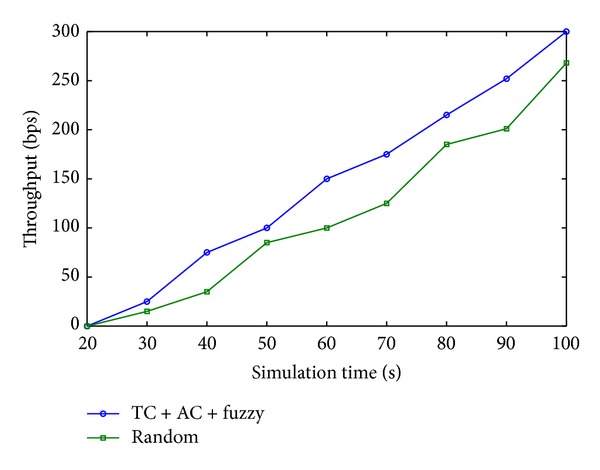
End-to-end throughput with *K* = 20 nodes.

Figures 4(a), 4(b), and 4(c) show the fuzzy inference for selection coordinator in face of jam attack.  Figure 5 shows energy effect of *E* = 3, *E* = 5, and *E* = 6 on the selection level for coordinator in face of jam attack.  Figure 6 shows that, by selecting coordinator, the throughput of networks is increased, for different selection coordinator methods for defending jam attack. Performance of the proposed algorithm shows that, by game selection and fuzzy inference, the throughput of networks is increased up to 300 bps (for a Zigbee network with 20 nodes) with TC + AC + Fuzzy rules, while the throughput of networks is increased up to 275 bps with random selection coordinator.

## 6. Conclusion

We have presented a coordinator selection scheme for ZWSNs to defend action from malicious nodes and minimize the cost of wireless transmission energy. By exploiting coordinator selection among multiple sensor nodes, the path security and reliability can be improved and the cost of data transmission can be reduced by selecting rules. We have formulated a game-theoretic model for joint dynamic defense and response strategy, taking into account the fact that each sensor node is rational to maximize its own payoff. The proposed game model is composed of two formulations, that is, a stochastic game for dynamic attack response and evolutionary game for coordinator selecting. The solutions of these games can achieve Nash equilibrium for the attack response strategy game.

## Figures and Tables

**Table 1:** The fuzzy rule matrix.

*E*_0_/*C*_0_	LO	HG
LO	LO	LO
ME	ME	ME
HG	ME	HG

**Table 2:** Simulation scenarios.

Parameters	Value
Protocols	AODV, Mac/802.15.4
Number of nodes	20
Simulation area	50 × 50
Traffic type	cbr, Poisson
Packet size	70 Bytes
Packets rate	250 k
Distance	5 m, 9 m, 10 m, 11 m, and 12 m
Simulation time	100 s
